# Supplementary material for: Evaluating research ethics committees in Vietnam and Laos: Results of a validated self-assessment tool
Source: PLoS One. 2024 Aug 22;19(8):e0309084. doi: 10.1371/journal.pone.0309084 (PMC11340885; doi:10.1371/journal.pone.0309084)
Supplement: S1 File — (PDF) [file pone.0309084.s001.pdf]

DukeHealth
Institutional Review Board

DUHS INSTITUTIONAL REVIEW BOARD DECLARATION OF ACTIVITY NOT MEETING THE DEFINITION OF RESEARCH

The DUHS IRB has determined that the following activity does not meet the definition of research as described in 45 CFR 46.102(d), 21 CFR 50.3(c) and 21 CFR 56.10(c) and satisfies the Privacy Rule as described in 45 CFR 164.514.

Protocol ID: Pro00115082
Reference ID: Pro00115082-INIT-1.0
Protocol Title: QI Project- IRB survey in SE Asia
Principal Investigator: Walter Lee

This IRB declaration is in effect from January 23, 2024 and does not expire. However, please be advised that any change to the proposed research will require re-review by the IRB.

DUHS Institutional Review Board
Suite 900 Erwin Square | 2200 West Main St | Durham, NC | 27705 | 919.668.5111
Federalwide Assurance No: FWA 00009025
