# Supplementary material for: Evaluating research ethics committees in Vietnam and Laos: Results of a validated self-assessment tool
Source: PLoS One. 2024 Aug 22;19(8):e0309084. doi: 10.1371/journal.pone.0309084 (PMC11340885; doi:10.1371/journal.pone.0309084)
Supplement: S2 File — (PDF) [file pone.0309084.s002.pdf]

Research Ethics Committee (REC) Quality Assurance Self-Assessment Tool

ORGANIZATIONAL ASPECTS (Maximum 54 POINTS)

What year was the REC established? _______					
1.	Is the REC subject to registration with a national authority? ___ Yes ___ No				**2 points**
2.	How often does the REC meet as a full committee to review research studies?				
	___ once/week	___ twice/month	___ once/month	___ every two months	
	___ other	___ has not yet met to review protocol			
**For meeting frequency equal or greater than once/month, 1 point**					
3.	Was the REC established under a high ranking authority (e.g., President's office, Ministry of Health, etc.)? ___ Yes ___ No				**5 points**
4.	Does the REC have written Standard Operating Procedures? __ Yes __ No				**5 points**
5.	Does the REC have a policy that outlines the process for appointing the REC Chair? ___ Yes ___ No				**2 points**
6.	Which of the following criteria are used to select the Chair of the REC? (Check all that apply.)				
	___ prior training in ethics				**1 point**
	___ publication in ethics				**1 point**
	___ prior research experience				**1 point**
	___ other (please describe) ______________________________				
7.	Does the REC have a policy that describes the process for appointing the members of the REC and details the membership requirements and the terms of appointment? ___ Yes ___ No				**2 points**

8.	Which of the following criteria are used to select REC members? (Check all that apply.)	
	___ prior training in ethics	**1 point**
	___ publication in ethics	**1 point**
	___ prior research experience	**1 point**
	___ other (please describe) ____	
9.	Does the REC have a policy for disclosure and management of potential conflicts of interest for the members of the REC? ___ Yes ___ No	**5 points**
10.	Does the REC have a policy for disclosure and management of potential conflicts of interest for members of the research team? ___ Yes ___ No	**5 points**
11.	Does the REC have a quality improvement (QI) program for itself? ___ Yes ___ No<br><br>If yes, describe what was done in the last year and any changes that were made as a result of the QI program.<br>____	**5 points**
12.	Does the institution/organization regularly evaluate the operations of the REC (e.g., budgetary needs, adequacy of material resources, adequacy of policies and procedures and practices, appropriateness of the membership given the research being reviewed, and documentation of the training requirements of the REC members)? ___ Yes ___ No	**5 points**
13.	Does the REC have a mechanism whereby enrolled research participants can file complaints or direct questions regarding human subjects protection issues? ___ Yes ___ No	**5 points**
	If yes, please describe the mechanism.<br>____	

14.	How are records of the REC stored?	**(1 point maximum)**
	____ Paper folders in a locked file cabinet	**1 point**
	____ Electronic in a password-protected computer	**1 point**
	____ On an open shelf ____ Other	
15.	Quorum: Does the REC require that there be a certain number of members present in order to make the meeting official to review protocols? ___ Yes ___ No	**5 points**

Research Ethics Committee (REC) Quality Assurance Self-Assessment Tool

MEMBERSHIP AND EDUCATIONAL TRAINING (Maximum 30 POINTS)

<table>
<tr><td>1.</td><td colspan="2">How many members are there on the REC? ____</td><td>If ≥ 5 members, 2 points</td></tr>
<tr><td>2.</td><td colspan="2">How many are women? _____ How many are men? ____</td><td></td></tr>
<tr><td colspan="4">If female/male gender ratio is between 0.4 and 0.6, then 2 points</td></tr>
<tr><td>3.</td><td colspan="2">Are any of the members not affiliated with the institution, that is, the member is not employed by the institution and is not related to a person who is employed? ___ Yes ___ No</td><td>2 points</td></tr>
<tr><td>4.</td><td colspan="2">Are any of the members considered to be a non-scientist? ___Yes ___ No (A Non-Scientific Member is any member who does not have a terminal degree in a medical or scientific field.)</td><td>2 points</td></tr>
<tr><td colspan="3">Please note that one member may fulfill both criteria of non-scientist and non-affiliated, in which case, please check Yes for both #3 and #4.</td><td></td></tr>
<tr><td>5.</td><td colspan="2">Is there a requirement that the REC Chair (or the designee who is in charge of running the committee) has any prior formal training in research ethics? ___ Yes ___ No</td><td>5 points</td></tr>
<tr><td></td><td colspan="2">If yes, what type of training is required? (Check all that apply.)</td><td></td></tr>
<tr><td></td><td>___ web-based training</td><td>___ workshop in research ethics</td><td></td></tr>
<tr><td></td><td>___ course</td><td>___ other (please describe) ____</td><td></td></tr>
</table>

6.	Does the institution require that REC members have training in research ethics in order to be a member of the REC? ___ Yes ___ No		**5 points**
	If yes, what type of training is required? (Check all that apply.)		
	___ web-based training	___ workshop in research ethics	
	___ course	___ other (please describe) ____	
7.	Does the institution require that investigators have training in research ethics in order to submit protocols for review by the REC? ___ Yes ___ No		**5 points**
	If yes, what type of training is required? (Check all that apply.)		
	___ web-based training	___ workshop in research ethics	
	___ lecture	___ course	
	___ other (please describe) ____		
8.	Does the REC conduct continuing education in research ethics for its members on a regular basis? ___ Yes ___ No		**5 points**
9.	Does the REC document the human subjects protection training received by its members? ___ Yes ___ No		**2 points**

Research Ethics Committee (REC) Quality Assurance Self-Assessment Tool

SUBMISSION ARRANGEMENTS AND MATERIALS (Maximum 12 POINTS)

**Submission Arrangements of Research Protocols**	**1 point each**	
**Item**	**Yes**	**No**
Does the REC publish guidelines for submission of applications for the review by the REC?		
Does the REC require investigators to use a specific application form for the submission of their protocols to the REC?		
Does the REC have an informed consent template to help guide investigators in the writing of their informed consent forms?		
Does the REC require approval and signature of the department chair (or another individual) of the research protocol prior to the submission?		
Does the REC require a deadline for investigators to submit protocols for full committee review?		

**Submission Materials**		
**Which of the following items are requested from the Principal Investigators when they submit their research protocol to the REC?**	**1 point each**	
**Item**	**Yes**	**No**
Full protocol		
Informed consent form		
Investigator's qualifications [e.g., CV, medical license(s), etc.]		
Conflict of interests disclosure forms for members of the research team		
Recruitment material (e.g., advertisements, signs, posters, etc.), if applicable		
Questionnaires/surveys that will be used in the research, if applicable		
Investigators' Drug Brochure or materials describing the nature of the drug being used in a clinical trial, if applicable		

Research Ethics Committee (REC) Quality Assurance Self-Assessment Tool

MINUTES (Maximum 13 POINTS)

**Does the REC maintain minutes of each meeting? ___Yes ___No**	**5 points**	
**If minutes are kept, please answer the following questions regarding the minutes.**	**1 point each**	
**Item**	**Yes**	**No**
Do the minutes reflect that members were asked whether they had a conflict of interest regarding any of the protocols to be discussed and indicate that such members did not participate in the decision making process of the relevant protocols?		
Do the minutes document that a quorum was present for all actions requiring a decision?		
Do the minutes document that all actions included at least one scientist in the review and participated in the decision making process?		
Do the minutes document that all actions included at least one non-scientist in the review who participated in the decision making process?		
Do the minutes document that all actions included at least one person who is not affiliated with the institution in the review and participated in the decision making process?		
Do the minutes record the name of REC members who abstained from the decision making process and provided the reason for abstention?		
Do the minutes record the name of REC members who were excused from the discussion and decision making process due to a conflict of interest?		
Do the minutes reflect, when applicable, a discussion of the controversial aspects of the research protocol?		

Research Ethics Committee (REC) Quality Assurance Self-Assessment Tool

POLICIES REFERRING TO REVIEW PROCEDURES (Maximum 11 POINTS)

**Policies Referring to Review Procedures**	**1 point each**	
**Item**	**Yes**	**No**
Does the REC have a policy regarding how protocols will be reviewed?		
Does the REC bring in a consultant when necessary to provide scientific or other relevant expertise for review of a particular protocol?		
Do REC members receive the protocol and other materials at a specified time prior to the meeting?		
Does the REC require that reviewers use a checklist to document their ethical assessment of the research submission?		
Does the REC have a policy on the conditions for expedited REC review?		
Does the REC have a policy on the conditions for when studies may qualify for exempt status?		
Does the REC determine the interval of continuing review based on the risk of the study?		
Does the REC have a policy for how decisions are made (e.g., consensus or a vote)?		
Are members asked at the beginning interest regarding any the meeting as to whether they had a conflict of the protocols to be discussed and indicate that such members did not participate in the decision on the relevant protocols?		
Does the REC have a policy for communicating a decision?		
Does the REC have a policy for follow-up review?		

REVIEW OF SPECIFIC PROTOCOL ITEMS (Maximum 43 POINTS)

**Scientific Design and Conduct of the Study**	**1 point each**	
**Item**	**Yes**	**No**
Does the REC review the suitability of the investigators' qualifications to conduct the study?		
Does the REC review the adequacy of the clinical site, including the supporting staff, available facilities, and emergency procedures?		
Does the REC take into account prior scientific reviews or do they review the appropriateness of the study design in relation to the objectives of the study, the statistical methodology, and the potential for addressing the objectives with the smallest number of research participants?		

Research Ethics Committee (REC) Quality Assurance Self-Assessment Tool

**Considerations of Risks and Benefits**	**1 point each**	
**Item**	**Yes**	**No**
Does the REC identify the different risks of the research protocol?		
Does the REC determine whether risks have been minimized?		
Does the REC determine whether the risks are greater than minimal risk based on a written definition of minimal risk?		
Does the REC evaluate the probable benefits of the research to the participants?		
Does the REC evaluate the importance of the knowledge to society that may reasonably be expected to result from the research?		
Does the REC evaluate whether the risks to research participants are reasonable in relation to any anticipated benefits to participants and the importance of the knowledge to be gained by society?		

**Selection of Research Participants**	**1 point each**	
**Item**	**Yes**	**No**
Does the REC review the methods to identify and recruit potential participants?		
Does the REC review recruitment processes to ensure that the selection of subjects will be equitable in regards to gender, religion, and ethnicity?		
Does the REC identify the potential of the research for enrolling participants who are likely to be vulnerable to coercion or undue influence (such as children, prisoners, persons with mental disabilities, or persons who are economically or educationally disadvantaged)?		
Does the REC consider the justification for including vulnerable populations in the research?		
Does the REC consider and require that additional safeguards be included in the study to protect the rights and welfare of the subjects?		
Does the REC consider the appropriateness of any financial or material incentives offered to participants for their participation in the research?		

**Privacy and Confidentiality**	**1 point each**	
**Item**	**Yes**	**No**
Does the REC preserve privacy by evaluating the setting in which participants are recruited?		
Does the REC evaluate the methods for protecting the confidentiality of the collected research data?		

Research Ethics Committee (REC) Quality Assurance Self-Assessment Tool

**Community Consultation**	**1 point each**	
**Item**	**Yes**	**No**
Does the REC review whether the potential benefits of the research are relevant to the health needs of the local community/country?		
Does the REC review whether any successful study product will be reasonably available to the concerned communities after the research?		
Does the REC review whether the community was consulted regarding the design and implementation of the research, if applicable?		

**Safety Monitoring and Adequacy of Insurance to Cover Research-Related Injury**	**1 point each**	
**Item**	**Yes**	**No**
Does the REC require, when appropriate, that the research plan include adequate provisions for monitoring the data collected to ensure the safety of subjects?		
Does the REC consider whether the sponsors of the research have adequate insurance to cover the treatments of injury related to the research?		

**Pediatric Research**	**1 point each**	
**Item**	**Yes**	**No**
Does the REC evaluate the need to obtain the child's assent?		

**Informed Consent**	**1 point each**	
**Item**	**Yes**	**No**
Does the REC review the process by which informed consent will be obtained (e.g., how do investigators identify potential subjects, where does the informed consent process take place, are potential subjects allowed to take the consent form home and given enough time to ask questions, etc.)?		
Does the REC review which members of the research team will approach potential participants for their informed consent?		
Does the REC ensure that the informed consent document is understandable to the subject population?<br>Suggested ways to assess the consent form might include:<br>• evaluate the reading level of the consent document<br>• have a community member read the consent form<br>• require investigators to assess subjects' understanding of the consent form		
Does the REC waive the requirement to obtain informed consent that is based on written criteria?		
Does the REC waive the requirement to have a written signature on the informed consent document that is based on written criteria?		

Research Ethics Committee (REC) Quality Assurance Self-Assessment Tool

**Basic Elements of Informed Consent**		
**Does the REC evaluate whether informed consent forms contain the following basic elements of informed consent?**	**1 point each**	
**Item**	**Yes**	**No**
A statement that the study involves research		
An explanation of the purposes of the research		
The expected duration of the subject's participation		
A description of the procedures to be followed		
Identification of any experimental procedures		
A description of any reasonably foreseeable risks or discomforts to the participant		
A description of any benefits to the participant or to others that might reasonably be expected from the research		
A disclosure of appropriate alternative procedures or courses of treatment, if any, that might be advantageous to the subject		
A statement describing the extent, if any, to which confidentiality of records identifying the participant will be maintained		
For research involving more than minimal risk, an explanation as to whether any medical treatments are available if injury occurs and, if so, what the treatments consist of or where further information may be obtained		
An explanation of whom to contact for answers to pertinent questions about research		
An explanation of whom to contact for answers to pertinent questions about research participants' rights		
A statement that participation is voluntary		
A statement that refusal to participate will involve no penalty or loss of benefits to which the subject is otherwise entitled		
A statement that participant may discontinue participation at any time without penalty or loss of benefits to which the participant is otherwise entitled		

Research Ethics Committee (REC) Quality Assurance Self-Assessment Tool

COMMUNICATING A DECISION (APPROVAL LETTER) Maximum 5 POINTS

Please answer the following questions regarding the approval letter sent to the PI. If no approval letter is sent to the investigator, please skip this section.

**Which of the following items are in the approval letter?**	**1 point each**	
**Item**	**Yes**	**No**
Provide an expiration date that is 1 year from the date of the convened REC meeting in which the study was approved.		
Require the investigators to submit to the REC as an amendment any changes that occur in the research plan; for example, change in investigators, change in drug doses, change in the sample size, etc.		
Require the investigators to promptly report to the REC any adverse events or unanticipated problems.		
Require the investigators to promptly report to the REC any protocol deviations.		
Require investigators to use the REC-approved informed consent form that is stamped with an expiration date.		

CONTINUING REVIEW (Maximum 16 POINTS)

**Does the REC request a continuing review report from the investigators on at least a yearly basis? ___Yes ___No**	**5 points**	
**If yes, which of the following items are requested in the continuing review report?**	**1 point each**	
**Item**	**Yes**	**No**
Number of subjects enrolled		
Gender and ethnic/religious breakdown of enrolled subjects		
Number of subjects withdrawn from the research by the investigators		
The reasons for withdrawal		
Number of subjects who dropped out of the research		
The reasons why subjects dropped out		
Verification that informed consent was obtained from all subjects and that all signed consent forms are on file		
Number and description of serious adverse events in the previous year (SAEs)		
List of any protocol violations or deviations		
Any safety monitoring reports		
If the study is completed, submit a final report describing the study results.		

Research Ethics Committee (REC) Quality Assurance Self-Assessment Tool

REC RESOURCES (Maximum 16 POINTS)

1.	Does the REC(s) have its own yearly budget? __ Yes __ No			**5 points**
	If yes, is there a budget for training of administrative staff and REC members? __ Yes __ No			**1 point**
2.	Please check below the physical resources of the REC (check all that apply):			**1 point each**
	__ access to a meeting room			
	__ access to a computer and printer			
	__ access to the internet			
	__ access to a facsimile			
	__ access to cabinets for storage of the protocol files			
3.	Does the REC have administrative staff assigned to the REC? __ Yes __ No			**5 points**
	If yes:	Is the person full-time?	__ Yes __ No	
		Is the person half-time?	__ Yes __ No	

Research Ethics Committee (REC) Quality Assurance Self-Assessment Tool

WORKLOAD OF THE REC (0 POINTS)

Average number of protocols reviewed annually? _______

Average number of clinical trials reviewed annually? _______

Average number of epidemiologic/observational studies reviewed annually? _______

After a brief review of three recent REC minutes, complete the following table with a specific number or N/A (not applicable).

**REC Workload Table**	**1st Meeting**	**2nd Meeting**	**3rd Meeting**
Duration of the meeting			
Number of new protocols reviewed by full committee			
Number of protocols disapproved			
Number of continuing review protocols approved by expedited review that were reported to the REC			
Number of continuing review protocols reviewed by full committee			
Number of amendments approved by expedited review that were reported to the REC			
Number of amendments reviewed by full committee			
Number of adverse reactions reviewed by full committee			
