# Supplementary material for: Evaluating research ethics committees in Vietnam and Laos: Results of a validated self-assessment tool
Source: PLoS One. 2024 Aug 22;19(8):e0309084. doi: 10.1371/journal.pone.0309084 (PMC11340885; doi:10.1371/journal.pone.0309084)
Supplement: S4 Dataset — (DOCX) [file pone.0309084.s006.docx]

ເຄື່ອງມືການປະເມີນຕົນເອງສຳລັບການຮັບປະກັນຄຸນນະພາບຂອງຄະນະກໍາມະການຈັນຍາບັນການຄົ້ນຄວ້າວິໄຈ (REC)

ດ້ານໂຄງຮ່າງການຈັດຕັ້ງ

REC ສ້າງຕັ້ງຂຶ້ນໃນປີໃດ? __2002_____

1. REC ຕ້ອງໄດ້ລົງທະບຽນນຳໜ່ວຍງານພາຍໃນປະເທດແມ່ນບໍ?

_✓__ ແມ່ນ ___ ບໍ່

2. REC ປະຊຸມເປັນຄະນະກໍາມະການເຕັມເພື່ອພິຈາລະນາການສຶກສາຄົ້ນຄ້ວາເລື້ອຍໆສໍ່າໃດ?

___ ໜຶ່ງຄັ້ງ/ອາທິດ ___ ສອງຄັ້ງ/ເດືອນ ___ ໜຶ່ງຄັ້ງ/ເດືອນ __✓_ ທຸກໆສອງເດືອນ

___ ອື່ນໆ ___ ຍັງບໍ່ທັນປະຊຸມກັນເພື່ອພິຈາລະນາໂຄງຮ່າງການຄົ້ນຄວ້າວິໄຈເທື່ອ

3. REC ໄດ້ຮັບການສ້າງຕັ້ງຂຶ້ນພາຍໃຕ້ໜ່ວຍງານລະດັບສູງ (ເຊັ່ນ: ສຳນັກງານປະທານປະເທດ, ກະຊວງສາທາລະນະສຸກ ແລະ ອື່ນໆ) ແມ່ນບໍ?

__✓_ ແມ່ນ ___ ບໍ່

4. REC ໄດ້ຂຽນລະບຽບຂັ້ນຕອນການປະຕິບັດງານມາດຕະຖານແມ່ນບໍ?

_✓_ ແມ່ນ __ ບໍ່

5. REC ມີນະໂຍບາຍທີ່ກໍານົດຂະບວນການແຕ່ງຕັ້ງປະທານ REC ແມ່ນບໍ?

_✓__ແມ່ນ ___ ບໍ່

6. ເກນເງື່ອນໄຂໃດຕໍ່ໄປນີ້ຖືກນໍາໃຊ້ເພື່ອເລືອກປະທານຂອງ REC? (ໝາຍເອົາທັງໝົດທີ່ກ່ຽວຂ້ອງ).

_✓__ ການຝຶກອົບຮົມດ້ານຈັນຍາບັນໃນເມື່ອກ່ອນ

_✓__ ການພິມເຜີຍແຜ່ໃນດ້ານຈັນຍາບັນ

_✓__ ປະສົບການດ້ານການຄົ້ນຄວ້າວິໄຈໃນເມື່ອກ່ອນ

_✓__ ອື່ນໆ (ກະລຸນາອະທິບາຍ) _____ຂົງເຂດວິຊາການສະເພາະ (ຊ່ຽວຊານສະເພາະ)______________

7. REC ມີນະໂຍບາຍທີ່ອະທິບາຍຂະບວນການແຕ່ງຕັ້ງສະມາຊິກຂອງ REC ແລະ ໃຫ້ລາຍລະອຽດກ່ຽວກັບຂໍ້ກຳນົດດ້ານການເປັນການສະມາຊິກ ແລະ ເງື່ອນໄຂການແຕ່ງຕັ້ງແມ່ນບໍ?

_✓__ ແມ່ນ ___ ບໍ່

8. ເກນເງື່ອນໄຂໃດຕໍ່ໄປນີ້ຖືກໃຊ້ເພື່ອເລືອກສະມາຊິກ REC? (ໝາຍເອົາທັງໝົດທີ່ກ່ຽວຂ້ອງ).

___ ການຝຶກອົບຮົມດ້ານຈັນຍາບັນໃນເມື່ອກ່ອນ

_✓__ ການພິມເຜີຍແຜ່ໃນດ້ານຈັນຍາບັນ

_✓__ ປະສົບການດ້ານການຄົ້ນຄວ້າວິໄຈໃນເມື່ອກ່ອນ

_✓__ ອື່ນໆ (ກະລຸນາອະທິບາຍ) _____ຊ່ຽວຊານສະເພາະ________________________________

9. REC ມີນະໂຍບາຍສໍາລັບການເປີດເຜີຍ ແລະ ການຄຸ້ມຄອງການຂັດແຍ່ງຜົນປະໂຫຍດທີ່ເປັນໄປໄດ້ສໍາລັບສະມາຊິກຂອງ REC ແມ່ນບໍ?

__✓_ ແມ່ນ ___ ບໍ່

10. REC ມີນະໂຍບາຍສໍາລັບການເປີດເຜີຍ ແລະ ການຄຸ້ມຄອງການຂັດແຍ້ງຜົນປະໂຫຍດທີ່ເປັນໄປໄດ້ຂອງສະມາຊິກທີມງານຄົ້ນຄ້ວາວິໄຈແມ່ນບໍ?

__✓_ ແມ່ນ ___ ບໍ່

11. REC ມີໂຄງການປັບປຸງຄຸນນະພາບ (QI) ສໍາລັບຕົວເອງແມ່ນບໍ?

___ ແມ່ນ _✓__ ບໍ່

ຖ້າແມ່ນ, ອະທິບາຍສິ່ງທີ່ໄດ້ເຮັດໃນປີທີ່ຜ່ານມາ ແລະ ການປ່ຽນແປງຕ່າງໆທີ່ໄດ້ເຮັດເປັນຜົນມາ

ຈາກໂຄງການ QI. _____________________________________

12. ສະຖາບັນ / ອົງການຈັດຕັ້ງປະເມີນການດໍາເນີນງານຂອງ REC ຢ່າງເປັນປົກກະຕິບໍ (ເຊັ່ນ: ຄວາມຕ້ອງການດ້ານງົບປະມານ, ຄວາມພຽງພໍຂອງຊັບພະຍາກອນທາງວັດຖຸ, ຄວາມພຽງພໍຂອງນະໂຍບາຍ ແລະ ລະບຽບຂັ້ນຕອນ ແລະ ການປະຕິບັດ, ຄວາມເຫມາະສົມຂອງການເປັນສະມາຊິກທີ່ໄດ້ຮັບການປະເມີນຄືນ ແລະ ການບັນທຶກຄວາມຕ້ອງການດ້ານການຝຶກອົບຮົມຂອງສະມາຊິກ REC ເປັນເອກະສານ)?

___ ແມ່ນ _✓__ ບໍ່

13. REC ມີກົນໄກທີ່ຜູ້ເຂົ້າຮ່ວມການຄົ້ນຄວ້າວິໄຈທີ່ລົງທະບຽນສາມາດຍື່ນຄໍາຮ້ອງທຸກ ຫຼື ຄໍາຖາມໂດຍກົງກ່ຽວກັບບັນຫາການປົກປ້ອງຜູ້ເຂົ້າຮ່ວມການທົດລອງວິໄຈທີ່ເປັນມະນຸດແມ່ນບໍ?

_✓__ ແມ່ນ ___ບໍ່

ຖ້າແມ່ນ, ກະລຸນາອະທິບາຍກົນໄກນັ້ນ.

___ທຸກການຄົ້ນຄວ້າ ເວລາທີ່ເຮັດໃບສະໝັກໃຈເຂົ້າຮ່ວມການສຶກສາ ແມ່ນໄດ້ລະບຸ ແລະ ໃສ່ເບີໂທຕິດຕໍ່ຫາໜ່ວຍງານຈັນຍາທໍາ ຫາກມີບັນຫາ ຫຼື ມີຄໍາຖາມສົງໄສແມ່ນສາມາດພົບພັນຜ່ານທາງອີເມລ ຫຼື ເບີໂທລະສັບໄດ້_____________

14. ບັນທຶກຂອງ REC ຖືກເກັບໄວ້ແນວໃດ?

__✓__ ໂຟນເດີເຈ້ຍຢູ່ໃນຕູ້ເອກະສານທີ່ມີກະແຈລັອກໄວ້

__✓__ ແບບເອເລັກໂຕຣນິກໃນຄອມພິວເຕີທີ່ມີລະຫັດຜ່ານປ້ອງກັນໄວ້

____ ຢູ່ເທິງຊັ້ນວາງເອກະສານແບບເປີດ

____ ອື່ນໆ

15. ອົງປະຊຸມ: REC ກຳນົດວ່າຕ້ອງມີສະມາຊິກຈຳນວນແນ່ນອນເຂົ້າຮ່ວມປະຊຸມຈິ່ງເຮັດໃຫ້ກອງປະຊຸມເປັນທາງການໃນການພິຈາລະນາໂຄງຮ່າງການຄົ້ນຄວ້າວິໄຈຕ່າງໆແມ່ນບໍ?

___ ແມ່ນ __✓_ ບໍ່

ການເປັນສະມາຊິກ ແລະ ການຝຶກອົບຮົມດ້ານການສຶກສາ

1. ມີສະມາຊິກຈັກຄົນຢູ່ໃນ REC? __15__

2. ມີແມ່ຍິງຈັກຄົນ? __10___ ມີຜູ້ຊາຍຈັກຄົນ? __5__

3. ມີສະມາຊິກໃດໆທີ່ບໍ່ກ່ຽວຂ້ອງກັບສະຖາບັນ, ນັ້ນແມ່ນ, ສະມາຊິກບໍ່ໄດ້ເປັນລູກຈ້າງຂອງ

ສະຖາບັນ ແລະ ບໍ່ກ່ຽວຂ້ອງກັບບຸກຄົນທີ່ເປັນລູກຈ້າງແມ່ນບໍ? ___ ແມ່ນ _✓__ ບໍ່

4. ມີສະມາຊິກໃດໆທີ່ຖືວ່າບໍ່ແມ່ນນັກວິທະຍາສາດບໍ? _✓__ແມ່ນ ___ ບໍ່

(**ສະມາຊິກທີ່ບໍ່ແມ່ນນັກວິທະຍາສາດ** ເປັນສະມາຊິກໃດໆກໍຕາມທີ່ບໍ່ມີລະດັບການສຶກສາໃນສາຂາການແພດ ຫຼື ວິທະຍາສາດ).

ກະລຸນາຮັບຊາບວ່າສະມາຊິກຄົນໜຶ່ງສາມາດຕອບສະໜອງທັງສອງເກນເງື່ອນໄຂທີ່ບໍ່ເປັນນັກວິທະຍາສາດ ແລະ ບໍ່ກ່ຽວຂ້ອງກັບສະຖາບັນ, ໃນກໍລະນີໃດ,

ກະລຸນາໝາຍເອົາ ແມ່ນ ສໍາລັບທັງສອງຂໍ້ #3 ແລະ #4.

5. ມີຂໍ້ກໍານົດວ່າປະທານ REC (ຫຼື ຜູ້ທີ່ໄດ້ຮັບການມອບໝາຍໃຫ້ຮັບຜິດຊອບນຳພາຄະນະກໍາມະການ) ຕ້ອງມີການຝຶກອົບຮົມທາງການໃນດ້ານຈັນຍາບັນການຄົ້ນຄວ້າວິໄຈມາກ່ອນແມ່ນບໍ? _✓__ ແມ່ນ ___ ບໍ່

ຖ້າແມ່ນ, ຕ້ອງມີການຝຶກອົບຮົມປະເພດໃດແດ່? (ໝາຍເອົາທັງໝົດທີ່ກ່ຽວຂ້ອງ).

___ ການຝຶກອົບຮົມຜ່ານເວັບ __✓_ ກອງປະຊຸມສຳມະນາກ່ຽວກັບຈັນຍາບັນການຄົ້ນຄວ້າວິໄຈ

___ ຫຼັກສູດ ___ ອື່ນໆ (ກະລຸນາອະທິບາຍ) __________________________________________________________

6. ສະຖາບັນກຳນົດໃຫ້ສະມາຊິກ REC ຕ້ອງມີການຝຶກອົບຮົມດ້ານຈັນຍາບັນການຄົ້ນຄວ້າວິໄຈຈິ່ງສາມາດເປັນສະມາຊິກຂອງ REC ໄດ້ແມ່ນບໍ?

___ ແມ່ນ _✓__ ບໍ່

ຖ້າແມ່ນ, ຕ້ອງມີການຝຶກອົບຮົມປະເພດໃດແດ່? (ໝາຍເອົາທັງໝົດທີ່ກ່ຽວຂ້ອງ).

___ ການຝຶກອົບຮົມຜ່ານເວັບ ___ ກອງປະຊຸມສຳມະນາກ່ຽວກັບຈັນຍາບັນການຄົ້ນຄວ້າວິໄຈ

___ ຫຼັກສູດ ___ ອື່ນໆ (ກະລຸນາອະທິບາຍ) __________________________________________________________

7. ສະຖາບັນກຳນົດໃຫ້ຜູ້ດຳເນີນການຄົ້ນຄວ້າວິໄຈຕ້ອງມີການຝຶກອົບຮົມດ້ານຈັນຍາບັນການຄົ້ນຄວ້າວິໄຈຈິ່ງສາມາດສົ່ງໂຄງຮ່າງການຄົ້ນຄວ້າວິໄຈໃຫ້ REC ພິຈາລະນາໄດ້ແມ່ນບໍ?

___ ແມ່ນ __✓_ ບໍ່

ຖ້າແມ່ນ, ຕ້ອງມີການຝຶກອົບຮົມປະເພດໃດແດ່? (ໝາຍເອົາທັງໝົດທີ່ກ່ຽວຂ້ອງ).

___ ການຝຶກອົບຮົມຜ່ານເວັບ ___ ກອງປະຊຸມສຳມະນາກ່ຽວກັບຈັນຍາບັນການຄົ້ນຄວ້າວິໄຈ

___ ການບັນຍາຍ ___ ຫຼັກສູດ

__________________ ອື່ນໆ (ກະລຸນາອະທິບາຍ) __________________________________________________________

8. REC ດໍາເນີນການສຶກສາແບບຕໍ່ເນື່ອງໃນດ້ານຈັນຍາບັນການຄົ້ນຄວ້າວິໄຈສໍາລັບສະມາຊິກຂອງຕົນຢ່າງເປັນປົກກະຕິແມ່ນບໍ?

_✓__ ແມ່ນ ___ ບໍ່

9. REC ບັນທຶກການຝຶກອົບຮົມກ່ຽວກັບການປົກປ້ອງຜູ້ເຂົ້າຮ່ວມການທົດລອງວິໄຈທີ່ສະມາຊິກຂອງຕົນໄດ້ຮັບໄວ້ເປັນເອກະສານແມ່ນບໍ?

_✓__ ແມ່ນ ___ ບໍ່

ການ​ຈັດວາງການ​ສົ່ງ ​ແລະ​ ເອກະສານ

REC ເຜີຍແຜ່ຂໍ້ແນະນໍາສຳລັບການສົ່ງຄຳຮ້ອງຂໍການພິຈາລະນາຈາກ REC ແມ່ນບໍ?

_✓__ ແມ່ນ ___ ບໍ່

REC ກຳນົດໃຫ້ຜູ້ດຳເນີນການຄົ້ນຄວ້າວິໄຈໃຊ້ແບບຟອມຄໍາຮ້ອງສະເພາະສໍາລັບການສົ່ງໂຄງຮ່າງການຄົ້ນຄວ້າວິໄຈຂອງເຂົາເຈົ້າໃຫ້ REC ແມ່ນບໍ?

_✓__ ແມ່ນ ___ ບໍ່

REC ມີແມ່ແບບການຍິນຍອມເຫັນດີແບບແຈ້ງໃຫ້ຮູ້ເພື່ອຊ່ວຍແນະນໍາຜູ້ສືບສວນໃນການຂຽນແບບຟອມການຍິນຍອມເຫັນດີແບບແຈ້ງໃຫ້ຮູ້ຂອງເຂົາເຈົ້າແມ່ນບໍ?

___ ແມ່ນ _✓__ ບໍ່

REC ກຳນົດໃຫ້ມີການອະນຸມັດ ແລະ ລາຍເຊັນຂອງປະທານພະແນກ (ຫຼື ບຸກຄົນອື່ນ) ຂອງໂຄງຮ່າງການຄົ້ນຄວ້າວິໄຈກ່ອນທີ່ຈະສົ່ງແມ່ນບໍ?

_✓__ ແມ່ນ ___ ບໍ່

REC ກໍານົດເສັ້ນຕາຍໃຫ້ຜູ້ດຳເນີນການຄົ້ນຄວ້າວິໄຈຕ້ອງສົ່ງໂຄງຮ່າງການຄົ້ນຄວ້າວິໄຈເພື່ອໃຫ້ຄະນະກຳມະການພິຈາລະນາແບບຄົບຖ້ວນແມ່ນບໍ?

_✓__ ແມ່ນ ___ ບໍ່

ມີການຂໍເອົາລາຍການເອກະສານໃດຕໍ່ໄປນີ້ຈາກຫົວໜ້າຜູ້ດຳເນີນການຄົ້ນຄວ້າວິໄຈເມື່ອເຂົາເຈົ້າສົ່ງ

ໂຄງຮ່າງການຄົ້ນຄວ້າວິໄຈຂອງເຂົາເຈົ້າໃຫ້ REC?

ໂຄງຮ່າງການຄົ້ນຄວ້າວິໄຈສະບັບເຕັມ

__✓_ ແມ່ນ ___ ບໍ່

ແບບຟອມການຍິນຍອມເຫັນດີແບບແຈ້ງໃຫ້ຮູ້

__✓_ ແມ່ນ ___ ບໍ່

ຄຸນສົມບັດຂອງຜູ້ດຳເນີນການຄົ້ນຄວ້າວິໄຈ [ເຊັ່ນ: ຊີວະປະຫວັດ, ໃບອະນຸຍາດທາງການແພດ ແລະ ອື່ນໆ]

_✓__ ແມ່ນ ___ ບໍ່

ແບບຟອມການເປີດເຜີຍການຂັດແຍ່ງຜົນປະໂຫຍດສໍາລັບສະມາຊິກຂອງທີມງານຄົ້ນຄ້ວາວິໄຈ

___ ແມ່ນ __✓_ ບໍ່

ເອກະສານການຮັບສະໝັກ (ເຊັ່ນ: ການໂຄສະນາ, ປ້າຍ, ໂປສເຕີ້ ແລະ ອື່ນໆ), ຖ້າມີ

___ ແມ່ນ _✓__ ບໍ່

ມີການຂໍເອົາລາຍການໃດຕໍ່ໄປນີ້ຈາກຜູ້ດຳເນີນການຄົ້ນຄວ້າວິໄຈເມື່ອເຂົາເຈົ້າສົ່ງໂຄງຮ່າງການຄົ້ນຄວ້າວິໄຈຂອງເຂົາເຈົ້າໃຫ້ REC?

ແບບສອບຖາມ/ແບບສຳຫຼວດທີ່ຈະໃຊ້ໃນການຄົ້ນຄວ້າວິໄຈ, ຖ້າມີ

_✓__ ແມ່ນ ___ ບໍ່

ແຜ່ນພັບໂຄສະນາຢາ ຫຼື ເອກະສານຂອງຜູ້ດຳເນີນການຄົ້ນຄວ້າວິໄຈທີ່ອະທິບາຍລັກສະນະຂອງຢາທີ່ໃຊ້ໃນການທົດລອງທາງຄລີນິກ, ຖ້າມີ

_✓__ ແມ່ນ ___ ບໍ່

ນາທີ

REC ຮັກສາບັນທຶກກອງປະຊຸມຂອງແຕ່ລະກອງປະຊຸມແມ່ນບໍ?

___ແມ່ນ ___ບໍ່

ຖ້າມີການເກັບຮັກສາບັນທຶກກອງປະຊຸມໄວ້, ກະລຸນາຕອບຄໍາຖາມຕໍ່ໄປນີ້ກ່ຽວກັບບັນທຶກກອງປະຊຸມ.

ບົດບັນກອງປະຊຸມສະແດງໃຫເ້ຫັນວ່າສະມາຊິກໄດ້ຖືກຖາມວ່າເຂົາເຈົ້າມີການຂັດແຍ້ງຜົນປະໂຫຍດກ່ຽວກັບໂຄງຮ່າງການຄົ້ນຄວ້າວິໄຈທີ່ຈະເວົ້າເຖິງຫຼືບໍ່ ແລະ ຊີ້ໃຫ້ເຫັນວ່າສະມາຊິກດັ່ງກ່າວບໍ່ໄດ້ເຂົ້າຮ່ວມໃນຂະບວນການຕັດສິນໃຈຂອງໂຄງຮ່າງການຄົ້ນຄວ້າວິໄຈທີ່ກ່ຽວຂ້ອງແມ່ນບໍ?

_✓__ ແມ່ນ ___ ບໍ່

ບົດບັນທຶກກອງປະຊຸມໄດ້ບັນທຶກວ່າມີອົງປະຊຸມຄົບຖ້ວນສຳລັບທຸກການດຳເນີນການທີ່ຕ້ອງມີການຕັດສິນໃຈແມ່ນບໍ?

_✓__ ແມ່ນ ___ ບໍ່

ບັນທຶກກອງປະຊຸມບັນທຶກວ່າການດຳເນີນການທັງໝົດລວມມີນັກວິທະຍາສາດຢ່າງໜ້ອຍໜຶ່ງຄົນຢູ່ໃນການພິຈາລະນາ ແລະ ເຂົ້າຮ່ວມໃນຂະບວນການຕັດສິນໃຈແມ່ນບໍ?

_✓__ ແມ່ນ ___ ບໍ່

ບັນທຶກກອງປະຊຸມບັນທຶກວ່າການດຳເນີນການທັງໝົດລວມມີຢ່າງໜ້ອຍໜຶ່ງຄົນທີ່ບໍ່ແມ່ນນັກວິທະຍາສາດຢູ່ໃນການພິຈາລະນາ ແລະ ເຂົ້າຮ່ວມໃນຂະບວນການຕັດສິນໃຈແມ່ນບໍ?

__✓_ ແມ່ນ ___ ບໍ່

ບັນທຶກກອງປະຊຸມບັນທຶກວ່າການດຳເນີນການທັງໝົດລວມມີຢ່າງໜ້ອຍໜຶ່ງຄົນທີ່ບໍ່ກ່ຽວຂ້ອງກັບສະຖາບັນຢູ່ໃນການພິຈາລະນາ ແລະ ເຂົ້າຮ່ວມໃນຂະບວນການຕັດສິນໃຈແມ່ນບໍ?

__✓_ ແມ່ນ ___ ບໍ່

ບົດບັນທຶກກອງປະຊຸມບັນທຶກຊື່ຂອງສະມາຊິກ REC ທີ່ງົດເຂົ້າຮ່ວມຂະບວນການຕັດສິນໃຈ ແລະ ໃຫ້ເຫດຜົນຂອງການງົດອອກສຽງແມ່ນບໍ?

___ ແມ່ນ _✓__ ບໍ່

ບົດບັນທຶກກອງປະຊຸມບັນທຶກຊື່ຂອງສະມາຊິກ REC ທີ່ບໍ່ເຂົ້າຮ່ວມການສົນທະນາ ແລະ ຂະບວນການຕັດສິນໃຈເນື່ອງການຂັດແຍ້ງຜົນປະໂຫຍດແມ່ນບໍ?

___ ແມ່ນ _✓__ ບໍ່

ເມື່ອກ່ຽວຂ້ອງ, ບົດບັນທຶກກອງປະຊຸມສະແດງໃຫ້ເຫັນການສົນທະນາກ່ຽວກັບດ້ານທີ່ຂັດແຍ່ງກັນຂອງໂຄງຮ່າງການຄົ້ນຄວ້າວິໄຈແມ່ນບໍ?

___ ແມ່ນ _✓__ ບໍ່

ນະໂຍບາຍທີ່ອ້າງເຖິງລະບຽບຂັ້ນຕອນການພິຈາລະນາ

REC ມີນະໂຍບາຍກ່ຽວກັບວ່າຈະພິຈາລະນາໂຄງຮ່າງການຄົ້ນຄວ້າວິໄຈແນວໃດແມ່ນບໍ?

_✓__ ແມ່ນ ___ ບໍ່

REC ໄດ້ນໍາເອົາທີ່ປຶກສາເຂົ້າມາໃນເວລາທີ່ຈໍາເປັນເພື່ອສະຫນອງຄວາມຊ່ຽວຊານດ້ານວິທະຍາສາດຫຼື ອື່ນໆທີ່ກ່ຽວຂ້ອງສໍາລັບການພິຈາລະນາໂຄງຮ່າງການຄົ້ນຄວ້າວິໄຈສະເພາະໃດໜຶ່ງແມ່ນບໍ?

__✓_ ແມ່ນ ___ ບໍ່

ສະມາຊິກ REC ໄດ້ຮັບໂຄງຮ່າງການຄົ້ນຄວ້າວິໄຈ ແລະ ເອກະສານອື່ນໆໃນເວລາທີ່ກໍານົດໄວ້ກ່ອນກອງປະຊຸມແມ່ນບໍ?

_✓__ ແມ່ນ ___ ບໍ່

REC ກຳນົດໃຫ້ຜູ້ພິຈາລະນາໂຄງຮ່າງການຄົ້ນຄວ້າວິໄຈຕ້ອງໃຊ້ລາຍການກວດສອບເພື່ອບັນທຶກການປະເມີນດ້ານຈັນຍາບັນຂອງເຂົາເຈົ້າຕໍ່ກັບການຍື່ນສະເຫນີໂຄງການຄົ້ນຄວ້າວິໄຈແມ່ນບໍ?

_✓__ ແມ່ນ ___ ບໍ່

REC ມີນະໂຍບາຍກ່ຽວກັບເງື່ອນໄຂສໍາລັບການພິຈາລະນາຂອງ REC ແບບເລັ່ງລັດແມ່ນບໍ?

_✓__ ແມ່ນ ___ ບໍ່

REC ມີນະໂຍບາຍກ່ຽວກັບເງື່ອນໄຂສໍາລັບເວລາທີ່ການສຶກສາວິໄຈອາດຈະມີຄຸນສົມບັດໄດ້ຮັບສະຖານະການເວັ້ນແມ່ນບໍ?

_✓__ ແມ່ນ ___ ບໍ່

REC ກໍານົດຊ່ວງເວລາຂອງການພິຈາລະນາແບບຕໍ່ເນື່ອງທີ່ໃສ່ຄວາມສ່ຽງຂອງການສຶກສາວິໄຈແມ່ນບໍ?

__✓_ ແມ່ນ ___ ບໍ່

REC ມີນະໂຍບາຍສໍາລັບການຕັດສິນໃຈແນວໃດ (ເຊັ່ນ: ການເອົາສຽງສ່ວນໃຫຍ່ ຫຼື ການລົງຄະແນນສຽງ) ແມ່ນບໍ?

__✓_ ແມ່ນ ___ ບໍ່

ມີການຖາມສະມາຊິກໃນຕອນເລີ່ມຕົ້ນກ່ຽວກັບກອງປະຊຸມໃດໆກ່ຽວກັບວ່າເຂົາເຈົ້າມີຂໍ້ຂັດແຍ່ງຂອງໂຄງຮ່າງການຄົ້ນຄວ້າວິໄຈທີ່ຈະປຶກສາຫາລືຫຼືບໍ່ ແລະ ຊີ້ໃຫ້ເຫັນວ່າສະມາຊິກດັ່ງກ່າວບໍ່ໄດ້ເຂົ້າຮ່ວມໃນການຕັດສິນໃຈກ່ຽວກັບໂຄງຮ່າງການຄົ້ນຄວ້າວິໄຈທີ່ກ່ຽວຂ້ອງແມ່ນບໍ?

_✓__ ແມ່ນ ___ ບໍ່

REC ມີນະໂຍບາຍສໍາລັບການສື່ສານແຈ້ງບອກການຕັດສິນໃຈແມ່ນບໍ?

___ ແມ່ນ _✓__ ບໍ່

REC ມີນະໂຍບາຍສຳລັບການພິຈາລະນາຕິດຕາມຜົນແມ່ນບໍ?

___ ແມ່ນ __✓_ ບໍ່

ການພິຈາລະນາລາຍການສະເພາະຂອງໂຄງຮ່າງການຄົ້ນຄວ້າວິໄຈ

ການອອກແບບທາງວິທະຍາສາດ ແລະ ການດຳເນີນການສຶກສາວິໄຈ

REC ໄດ້ພິຈາລະນາຄວາມເຫມາະສົມດ້ານຄຸນວຸດທິຂອງຜູ້ດຳເນີນການວິໄຈທີ່ຈະດຳເນີນການສຶກສາວິໄຈດັ່ງກ່າວແມ່ນບໍ?

_✓__ ແມ່ນ ___ ບໍ່

REC ໄດ້ພິຈາລະນາຄວາມພຽງພໍຂອງສະຖານທີ່ທາງຄລີນິກ, ລວມທັງພະນັກງານສະໜັບສະໜຸນ, ສິ່ງອຳນວຍຄວາມສະດວກທີ່ມີ ແລະ ລະບຽບຂັ້ນຕອນສຳລັບເຫດສຸກເສີນແມ່ນບໍ?

__✓_ ແມ່ນ ___ ບໍ່

REC ໄດ້ຄຳນຶງເຖິງການພິຈາລະນາທາງວິທະຍາສາດໃນເມື່ອກ່ອນ ຫຼື ເຂົາເຈົ້າໄດ້ພິຈາລະນາຄວາມເໝາະສົມຂອງການອອກແບບການສຶກສາວິໄຈທີ່ກ່ຽວຂ້ອງກັບຈຸດປະສົງຂອງການສຶກສາວິໄຈ, ວິທີການທາງສະຖິຕິ ແລະ ຄວາມເປັນໄປໄດ້ສຳລັບການແກ້ໄຂຈຸດປະສົງດ້ວຍຜູ້ເຂົ້າຮ່ວມການຄົ້ນຄວ້າວິໄຈຈຳນວນທີ່ໜ້ອຍໜຶ່ງ.

__✓_ ແມ່ນ ___ ບໍ່

REC ລະບຸຄວາມສ່ຽງຕ່າງໆຂອງໂຄງຮ່າງການຄົ້ນຄວ້າວິໄຈແມ່ນບໍ?

__✓_ ແມ່ນ ___ ບໍ່

REC ກໍານົດວ່າໄດ້ມີການຈຳກັດໃຫ້ມີຄວາມສ່ຽງໜ້ອຍສຸດແລ້ວແມ່ນຫຼືບໍ່?

__✓_ ແມ່ນ ___ ບໍ່

REC ກໍານົດວ່າຄວາມສ່ຽງແມ່ນຫຼາຍກວ່າຄວາມສ່ຽງໜ້ອຍສຸດຫຼືບໍ່ ອີງໃສ່ຄຳນິຍາມເປັນລາຍລັກອັກສອນຂອງຄວາມສ່ຽງໜ້ອຍສຸດ?

__✓_ ແມ່ນ ___ ບໍ່

REC ປະເມີນຜົນປະໂຫຍດທີ່ເປັນໄປໄດ້ຂອງການຄົ້ນຄວ້າວິໄຈຕໍ່ກັບຜູ້ເຂົ້າຮ່ວມແມ່ນບໍ?

_✓__ ແມ່ນ ___ ບໍ່

REC ປະເມີນຄວາມສໍາຄັນຂອງຄວາມຮູ້ຕໍ່ກັບສັງຄົມທີ່ຄາດຢ່າງສົມເຫດສົມຜົນວ່າຈະໄດ້ຈາກການຄົ້ນຄວ້າວິໄຈດັ່ງກ່າວແມ່ນບໍ?

__✓_ ແມ່ນ ___ ບໍ່

REC ປະເມີນວ່າຄວາມສ່ຽງຕໍ່ຜູ້ເຂົ້າຮ່ວມການຄົ້ນຄວ້າວິໄຈແມ່ນສົມເຫດສົມຜົນຫຼືບໍ່ ທຽບກັບຜົນປະໂຫຍດທີ່ຄາດວ່າຈະມີຕໍ່ຜູ້ເຂົ້າຮ່ວມ ແລະ ຄວາມສໍາຄັນຂອງຄວາມຮູ້ທີ່ສັງຄົມຈະໄດ້ຮັບ?

__✓_ ແມ່ນ ___ ບໍ່

ການເລືອກຜູ້ເຂົ້າຮ່ວມການຄົ້ນຄວ້າວິໄຈ

REC ໄດ້ພິຈາລະນາວິທີການກໍານົດ ແລະ ຄັດເລືອກຜູ້ເຂົ້າຮ່ວມທີ່ເປັນໄປໄດ້ແມ່ນບໍ?

_✓__ ແມ່ນ ___ ບໍ່

REC ໄດ້ພິຈາລະນາຂະບວນການຮັບສະໝັກເພື່ອຮັບປະກັນວ່າການຄັດເລືອກຜູ້ເຂົ້າຮ່ວມການທົດລອງວິໄຈຈະທົ່ວເຖິງທາງດ້ານບົດບາດຍິງຊາຍ, ສາດສະໜາ ແລະ ຊົນເຜົ່າແມ່ນບໍ?

___ ແມ່ນ _✓__ ບໍ່

REC ໄດ້ລະບຸຄວາມເປັນໄປໄດ້ຂອງການຄົ້ນຄວ້າວິໄຈສໍາລັບການລົງທະບຽນຜູ້ເຂົ້າຮ່ວມທີ່ເປັນໄປໄດ້ວ່າອາດຈະສ່ຽງຕໍ່ການບີບບັງຄັບ ຫຼື ອິດທິພົນທີ່ບໍ່ເຫມາະສົມ (ເຊັ່ນ: ເດັກນ້ອຍ, ນັກໂທດ, ຄົນພິການທາງຈິດ ຫຼື ບຸກຄົນທີ່ດ້ອຍໂອກາດທາງດ້ານເສດຖະກິດ ຫຼື ການສຶກສາ) ແມ່ນບໍ?

_✓__ ແມ່ນ ___ ບໍ່

REC ໄດ້ພິຈາລະນາເຫດຜົນສໍາລັບການລວມເອົາປະຊາກອນທີ່ມີຄວາມສ່ຽງເຂົ້າໃນການຄົ້ນຄວ້າວິໄຈບໍ?

__✓_ ແມ່ນ ___ ບໍ່

REC ພິຈາລະນາ ແລະ ກຳນົດໃຫ້ລວມເອົາການປົກປ້ອງເພີ່ມເຕີມເຂົ້າໃນການສຶກສາວິໄຈເພື່ອປົກປ້ອງສິດທິ ແລະ ສະຫວັດດີການຂອງຜູ້ເຂົ້າຮ່ວມການທົດລອງວິໄຈບໍ?

__✓_ ແມ່ນ ___ ບໍ່

ການເລືອກຜູ້ເຂົ້າຮ່ວມການຄົ້ນຄວ້າວິໄຈ

REC ພິຈາລະນາຄວາມເຫມາະສົມຂອງສິ່ງຈູງໃຈດ້ານການເງິນ ຫຼື ວັດຖຸທີ່ສະເຫນີໃຫ້ຜູ້ເຂົ້າຮ່ວມສໍາລັບການມີສ່ວນຮ່ວມໃນການຄົ້ນຄວ້າວິໄຈຂອງເຂົາເຈົ້າບໍ?

__✓_ ແມ່ນ ___ ບໍ່

ຄວາມເປັນສ່ວນຕົວ ແລະ ຄວາມລັບ

REC ຮັກສາຄວາມເປັນສ່ວນຕົວໂດຍການປະເມີນສະພາບການທີ່ຜູ້ເຂົ້າຮ່ວມໄດ້ຮັບການເລືອກບໍ?

_✓__ ແມ່ນ ___ ບໍ່

REC ປະເມີນວິທີການປົກປ້ອງຄວາມລັບຂອງຂໍ້ມູນການຄົ້ນຄວ້າວິໄຈທີ່ເກັບເອົາບໍ?

_✓__ ແມ່ນ ___ ບໍ່

ການປຶກສາຫາລືຊຸມຊົນ

REC ພິຈາລະນາວ່າຜົນປະໂຫຍດທີ່ເປັນໄປໄດ້ຂອງການຄົ້ນຄວ້າວິໄຈແມ່ນກ່ຽວຂ້ອງກັບຄວາມຕ້ອງການດ້ານສຸຂະພາບຂອງຊຸມຊົນ / ປະເທດທ້ອງຖິ່ນແມ່ນຫຼືບໍ່?

_✓__ ແມ່ນ ___ ບໍ່

REC ພິຈາລະນາວ່າຜະລິດຕະພັນຈາກການສຶກສາວິໄຈທີ່ປະສົບຜົນສໍາເລັດຕ່າງໆຈະມີໃຫ້ຢ່າງສົມເຫດສົມຜົນແກ່ຊຸມຊົນທີ່ກ່ຽວຂ້ອງຫຼັງຈາກການຄົ້ນຄວ້າວິໄຈຫຼືບໍ່?

__✓_ ແມ່ນ ___ ບໍ່

REC ພິຈາລະນາວ່າຊຸມຊົນໄດ້ຮັບການປຶກສາຫາລືກ່ຽວກັບການອອກແບບ ແລະ ການຈັດຕັ້ງປະຕິບັດການຄົ້ນຄວ້າວິໄຈຫຼືບໍ່, ຖ້າກ່ຽວຂ້ອງ?

___ ແມ່ນ __✓_ ບໍ່

ການຕິດຕາມຄວາມປອດໄພ ແລະ ຄວາມພຽງພໍຂອງປະກັນໄພທີ່ຈະກວມເອົາການບາດເຈັບທີ່ກ່ຽວຂ້ອງກັບການຄົ້ນຄວ້າວິໄຈ

ໃນເວລາທີ່ເໝາະສົມ, REC ກຳນົດວ່າແຜນການຄົ້ນຄ້ວາວິໄຈຕ້ອງລວມມີຂໍ້ກໍານົດທີ່ພຽງພໍສໍາລັບການຕິດຕາມກວດກາຂໍ້ມູນທີ່ເກັບເອົາເພື່ອຮັບປະກັນຄວາມປອດໄພຂອງຜູ້ເຂົ້າຮ່ວມການທົດລອງວິໄຈ?

__✓_ ແມ່ນ ___ ບໍ່

REC ພິຈາລະນາວ່າຜູ້ອຸປະຖຳການຄົ້ນຄວ້າວິໄຈມີການປະກັນໄພທີ່ພຽງພໍເພື່ອຄຸ້ມຄອງການປິ່ນປົວການບາດເຈັບທີ່ກ່ຽວຂ້ອງກັບການຄົ້ນຄວ້າວິໄຈຫຼືບໍ່?

__✓_ ແມ່ນ ___ ບໍ່

ການຄົ້ນຄວ້າວິໄຈແພດເດັກ

REC ປະເມີນຄວາມຕ້ອງການໄດ້ຮັບການຍິນຍອມຈາກເດັກບໍ?

__✓_ ແມ່ນ ___ ບໍ່

ການຍິນຍອມເຫັນດີແບບແຈ້ງໃຫ້ຮູ້

REC ພິຈາລະນາຂະບວນການທີ່ຈະມີການຂໍເອົາການຍິນຍອມເຫັນດີແບບແຈ້ງໃຫ້ຮູ້ (ເຊັ່ນ: ຜູ້ດຳເນີນການຄົ້ນຄວ້າວິໄຈລະບຸຕົວຜູ້ເຂົ້າຮ່ວມທົດລອງວິໄຈແນວໃດ, ຂະບວນການຍິນຍອມເຫັນດີເກີດຂື້ນຢູ່ບ່ອນໃດ, ຜູ້ເຂົ້າຮ່ວມທົດລອງວິໄຈໄດ້ຮັບອະນຸຍາດໃຫ້ເອົາແບບຟອມການຍິນຍອມກັບບ້ານ ແລະ ໄດ້ຮັບເວລາພຽງພໍເພື່ອຖາມຄໍາຖາມ ແລະ ອື່ນໆ) ບໍ?

__✓_ ແມ່ນ ___ ບໍ່

REC ພິຈາລະນາວ່າສະມາຊິກທີມຄົ້ນຄວ້າວິໄຈຄົນໃດຈະເຂົ້າຫາຜູ້ເຂົ້າຮ່ວມທີ່ເປັນໄປໄດ້ເພື່ອຂໍການຍິນຍອມເຫັນດີແບບແຈ້ງໃຫ້ຮູ້ກ່ອນຂອງເຂົາເຈົ້າບໍ?

_✓__ ແມ່ນ ___ ບໍ່

REC ຮັບປະກັນວ່າເອກະສານການຍິນຍອມເຫັນດີແບບແຈ້ງໃຫ້ຮູ້ສາມາດເຂົ້າໃຈໄດ້ຕໍ່ກັບກຸ່ມຜູ້ເຂົ້າຮ່ວມທົດລອງວິໄຈບໍ?

_✓__ ແມ່ນ___ ບໍ່

ການຍິນຍອມເຫັນດີແບບແຈ້ງໃຫ້ຮູ້

ວິທີການທີ່ແນະນໍາເພື່ອປະເມີນແບບຟອມການຍິນຍອມເຫັນດີອາດຈະປະກອບມີ:

ປະເມີນລະດັບການອ່ານຂອງເອກະສານການຍິນຍອມ

ໃຫ້ສະມາຊິກຊຸມຊົນອ່ານແບບຟອມການຍິນຍອມ

ກຳນົດໃຫ້ຜູ້ດຳເນີນການຄົ້ນຄວ້າວິໄຈປະເມີນຄວາມເຂົ້າໃຈແບບຟອມການຍິນຍອມເຫັນດີຂອງຜູ້ເຂົ້າຮ່ວມທົດລອງວິໄຈ.

REC ຍົກເວັ້ນຂໍ້ກຳນົດທີ່ຕ້ອງໄດ້ຮັບຂໍ້ມູນການຍິນຍອມເຫັນດີແບບແຈ້ງໃຫ້ຮູ້ທີ່ອີງໃສ່ເກນເງື່ອນໄຂທີ່ຂຽນໄວ້ບໍ?

__✓_ ແມ່ນ ___ ບໍ່

REC ຍົກເວັ້ນຂໍ້ກໍານົດໃຫ້ມີລາຍເຊັນເປັນລາຍລັກອັກສອນໃນເອກະສານການຍິນຍອມເຫັນດີແບບແຈ້ງໃຫ້ຮູ້ທີ່ທີ່ອີງໃສ່ເກນເງື່ອນໄຂທີ່ຂຽນໄວ້ບໍ?

_✓__ ແມ່ນ ___ ບໍ່

ອົງປະກອບພື້ນຖານຂອງການຍິນຍອມເຫັນດີແບບແຈ້ງໃຫ້ຮູ້

REC ປະເມີນວ່າແບບຟອມການຍິນຍອມເຫັນດີແບບແຈ້ງໃຫ້ຮູ້ມີອົງປະກອບພື້ນຖານຕໍ່ໄປນີ້ຂອງການຍິນຍອມເຫັນດີແບບແຈ້ງໃຫ້ຮູ້ຫຼືບໍ່?

ຄຳຖະແຫຼງວ່າການສຶກສາແມ່ນກ່ຽວຂ້ອງກັບການຄົ້ນຄວ້າວິໄຈ

_✓__ ແມ່ນ ___ ບໍ່

ຄໍາອະທິບາຍຈຸດປະສົງຂອງການຄົ້ນຄວ້າວິໄຈ

_✓__ ແມ່ນ ___ ບໍ່

ໄລຍະເວລາການເຂົ້າຮ່ວມທີ່ຄາດໄວ້ຂອງຜູ້ເຂົ້າຮ່ວມທົດລອງວິໄຈ

_✓__ ແມ່ນ ___ ບໍ່

ລາຍລະອຽດຂອງລະບຽບຂັ້ນຕອນທີ່ຕ້ອງປະຕິບັດຕາມ

_✓__ ແມ່ນ ___ ບໍ່

ການລະບຸລະບຽບຂັ້ນຕອນການທົດລອງຕ່າງໆ

_✓__ ແມ່ນ ___ ບໍ່

ລາຍ​ລະ​ອຽດ​ກ່ຽວ​ກັບ​ຄວາມ​ສ່ຽງ ຫຼື ຄວາມບໍ່ສະບາຍຕົວ​ທີ່​ຄາດການໄດ້ຢ່າງສົມ​ເຫດ​ສົມ​ຜົນຂອງຜູ້ເຂົ້າຮ່ວມ

_✓__ ແມ່ນ ___ ບໍ່

ລາຍລະອຽດຂອງຜົນປະໂຫຍດຕ່າງໆຕໍ່ກັບຜູ້ເຂົ້າຮ່ວມ ຫຼື ຄົນອື່ນທີ່ອາດຈະຄາດການໄດ້ຢ່າງສົມເຫດສົມຜົນວ່າຈະໄດ້ຮັບຈາກການຄົ້ນຄວ້າວິໄຈ

__✓_ ແມ່ນ ___ ບໍ່

ການເປີດເຜີຍຂັ້ນຕອນ ຫຼື ຫຼັກສູດການປິ່ນປົວທາງເລືອກທີ່ເຫມາະສົມ, ຖ້າມີ, ທີ່ອາດຈະເປັນປະໂຫຍດຕໍ່ກັບຜູ້ເຂົ້າຮ່ວມທົດລອງວິໄຈ

_✓__ ແມ່ນ ___ ບໍ່

ຄຳຖະແຫຼງທີ່ອະທິບາຍເຖິງຂອບເຂດ, ຖ້າມີ, ທີ່ຈະຮັກສາຄວາມລັບຂອງບັນທຶກທີ່ລະບຸຕົວຕົນຂອງຜູ້ເຂົ້າຮ່ວມ

__✓_ ແມ່ນ ___ ບໍ່

ສໍາລັບການຄົ້ນຄວ້າວິໄຈທີ່ກ່ຽວພັນກັບຫຼາຍກວ່າຄວາມສ່ຽງຫນ້ອຍສຸດ, ຄໍາອະທິບາຍກ່ຽວກັບວ່າຈະມີການປິ່ນປົວທາງການແພດໃຫ້ຫຼືບໍ່ຖ້າເກີດການບາດເຈັບ ແລະ, ຖ້າມີ, ການປິ່ນປົວປະກອບມີຫຍັງແດ່ ຫຼື ຈະສາມາດຂໍເອົາຂໍ້ມູນເພີ່ມເຕີມໄດ້ຈາກໃສ

_✓__ ແມ່ນ ___ ບໍ່

ຄໍາອະທິບາຍກ່ຽວກັບວ່າຕ້ອງຕິດຕໍ່ຫາໃຜເພື່ອຂໍຄຳຕອບສໍາລັບຄໍາຖາມທີ່ກ່ຽວຂ້ອງກັບການຄົ້ນຄວ້າວິໄຈ

__✓_ ແມ່ນ ___ ບໍ່

ຄໍາອະທິບາຍກ່ຽວກັບວ່າຈະຕິດຕໍ່ຫາໃຜເພື່ອເອົາຄຳຕອບສຳລັບຄໍາຖາມທີ່ກ່ຽວຂ້ອງກັບສິດທິຂອງຜູ້ເຂົ້າຮ່ວມການຄົ້ນຄວ້າວິໄຈ

__✓_ ແມ່ນ ___ ບໍ່

ຄຳຖະແຫຼງວ່າການເຂົ້າຮ່ວມແມ່ນຄວາມສະໝັກໃຈ

__✓_ ແມ່ນ ___ ບໍ່

ຄຳຖະແຫຼງວ່າການປະຕິເສດບໍ່ເຂົ້າຮ່ວມຈະບໍ່ຖືກລົງໂທດ ຫຼື ສູນເສຍຜົນປະໂຫຍດໃດໆທີ່ຜູ້ເຂົ້າຮ່ວມທົດລອງວິໄຈມີສິດໄດ້ຮັບ

_✓__ ແມ່ນ ___ ບໍ່

ຄຳຖະແຫຼງວ່າຜູ້ເຂົ້າຮ່ວມອາດຈະຢຸດເຊົາການເຂົ້າຮ່ວມໄດ້ທຸກເວລາໂດຍບໍ່ມີການລົງໂທດ ຫຼື ການສູນເສຍຜົນປະໂຫຍດທີ່ຜູ້ເຂົ້າຮ່ວມມີສິດໄດ້ຮັບ

_✓__ ແມ່ນ ___ ບໍ່

ການ​ສື່​ສານ​ແຈ້ງບອກການ​ຕັດ​ສິນ​ໃຈ (ໜັງສື​ອະ​ນຸ​ມັດ​)

ກະລຸນາຕອບຄຳຖາມຕໍ່ໄປນີ້ກ່ຽວກັບໜັງສືອະນຸມັດທີ່ສົ່ງໃຫ້ PI. ຖ້າບໍ່ມີ

ໜັງສືອະນຸມັດຖືກສົ່ງໄປໃຫ້ຜູ້ດຳເນີນການຄົ້ນຄວ້າວິໄຈ, ກະລຸນາຂ້າມພາກນີ້ໄປ.

ລາຍການໃດຕໍ່ໄປນີ້ຢູ່ໃນໜັງສືອະນຸມັດ?

ໃຫ້ວັນທີຫມົດອາຍຸທີ່ແມ່ນ 1 ປີນັບຈາກວັນທີຂອງກອງປະຊຸມ REC ທີ່ໄດ້ປະຊຸມອະນຸມັດການສຶກສາວິໄຈ.

_✓__ ແມ່ນ ___ ບໍ່

ກຳນົດໃຫ້ຜູ້ດຳເນີນການຄົ້ນຄວ້າວິໄຈສົ່ງໃຫ້ REC ເປັນການດັດແກ້ ການປ່ຽນແປງຕ່າງໆທີ່ເກີດຂຶ້ນໃນແຜນການຄົ້ນຄ້ວາວິໄຈ; ຕົວຢ່າງ, ການປ່ຽນແປງຜູ້ດຳເນີນການຄົ້ນຄວ້າວິໄຈ, ການປ່ຽນແປງປະລິມານຢາ, ການປ່ຽນແປງຂະຫນາດຕົວຢ່າງ ແລະ ອື່ນໆ.

_✓__ ແມ່ນ ___ ບໍ່

ກຳນົດໃຫ້ຜູ້ດຳເນີນການຄົ້ນຄວ້າວິໄຈລາຍງານເຫດການທາງລົບ ຫຼືບັນຫາທີ່ບໍ່ຄາດໄວ້ໃຫ້ REC ທັນທີ.

_✓__ ແມ່ນ ___ ບໍ່

ກຳນົດໃຫ້ຜູ້ດຳເນີນການຄົ້ນຄວ້າວິໄຈລາຍງານການຜິດບ່ຽງໄປຈາກໂຄງຮ່າງການຄົ້ນຄວ້າວິໄຈຕ່າງໆໃຫ້ REC ໃນທັນທີ.

__✓_ ແມ່ນ ___ ບໍ່

ກຳນົດໃຫ້ຜູ້ດຳເນີນການຄົ້ນຄວ້າວິໄຈໃຊ້ແບບຟອມການຍິນຍອມເຫັນດີແບບແຈ້ງໃຫ້ຮູ້ທີ່ໄດ້ຮັບອະນຸມັດຈາກ REC ທີ່ສະແຕມວັນທີຫມົດອາຍຸ.

_✓__ ແມ່ນ ___ ບໍ່

ການພິຈາລະນາແບບຕໍ່ເນື່ອງ

REC ກຳນົດໃຫ້ມີບົດລາຍງານການພິຈາລະນາແບບຕໍ່ເນື່ອງຈາກຜູ້ດຳເນີນການຄົ້ນຄວ້າວິໄຈຢ່າງຫນ້ອຍທຸກໆປີບໍ?

___ແມ່ນ ___ບໍ່

ຖ້າແມ່ນ, ມີການຂໍເອົາລາຍການໃດຕໍ່ໄປນີ້ໃນບົດລາຍງານການພິຈາລະນາແບບຕໍ່ເນື່ອງ?

ຈຳນວນຜູ້ເຂົ້າຮ່ວມທົດລອງວິໄຈທີ່ລົງທະບຽນ

__✓_ ແມ່ນ ___ ບໍ່

ລາຍລະອຽດດ້ານເພດ ແລະ ຊົນເຜົ່າ/ສາສະໜາຂອງຜູ້ເຂົ້າຮ່ວມທົດລອງວິໄຈທີ່ລົງທະບຽນ

__✓_ ແມ່ນ ___ ບໍ່

ຈໍານວນຜູ້ເຂົ້າຮ່ວມທົດລອງວິໄຈທີ່ຜູ້ດຳເນີນການຄົ້ນຄວ້າວິໄຈຖອນອອກຈາກການຄົ້ນຄວ້າວິໄຈ

_✓__ ແມ່ນ ___ ບໍ່

ເຫດຜົນສໍາລັບການຖອນອອກ

__✓_ ແມ່ນ ___ ບໍ່

ຈໍານວນຜູ້ເຂົ້າຮ່ວມທົດລອງວິໄຈທີ່ອອກຈາກການຄົ້ນຄວ້າວິໄຈ

_✓__ ແມ່ນ ___ ບໍ່

ເຫດຜົນຍ້ອນຫຍັງຈິ່ງອອກໄປ

_✓__ ແມ່ນ ___ ບໍ່

ການກວດຢັ້ງຢືນວ່າການຍິນຍອມເຫັນດີແບບແຈ້ງໃຫ້ຮູ້ແມ່ນໄດ້ຮັບຈາກຜູ້ເຂົ້າຮ່ວມທົດລອງວິໄຈທຸກຄົນ ແລະ ແບບຟອມການຍິນຍອມເຫັນດີທີ່ມີລາຍເຊັນທັງໝົດແມ່ນຢູ່ໃນແຟ້ມເອກະສານ

_✓__ ແມ່ນ ___ ບໍ່

ຈໍາ​ນວນ ​ແລະ ​ລາຍ​ລະ​ອຽດ​ຂອງ​ເຫດ​ການ​ທາງ​ລົບ​ທີ່​ຮ້າຍ​ແຮງ​ໃນ​ປີ​ທີ່​ຜ່ານ​ມາ (SAE​)

_✓__ ແມ່ນ ___ ບໍ່

ລາຍ​ຊື່​ຂອງ​ການ​ລະ​ເມີດໂຄງຮ່າງການຄົ້ນຄວ້າວິໄຈ ຫຼື ການຜິດບ່ຽງຕ່າງໆ

__✓_ ແມ່ນ ___ ບໍ່

ບົດລາຍງານການຕິດຕາມຄວາມປອດໄພຕ່າງໆ

__✓_ ແມ່ນ ___ ບໍ່

ຖ້າການສຶກສາວິໄຈສໍາເລັດ, ສົ່ງບົດລາຍງານສຸດທ້າຍທີ່ອະທິບາຍຜົນການສຶກສາວິໄຈ.

__✓_ ແມ່ນ ___ ບໍ່

ຊັບພະຍາກອນຂອງ REC

1. REC ມີງົບປະມານປະຈຳປີຂອງຕົນເອງແມ່ນບໍ? __ ແມ່ນ _✓_ ບໍ່

ຖ້າແມ່ນ, ມີງົບປະມານສໍາລັບການຝຶກອົບຮົມພະນັກງານບໍລິຫານ ແລະ ສະມາຊິກ REC ແມ່ນບໍ?

__ ແມ່ນ __ ບໍ່

2. ກະລຸນາກວດເບິ່ງຊັບພະຍາກອນທາງວັດຖຸຂອງ REC ຂ້າງລຸ່ມ (ໝາຍເອົາທັງໝົດທີ່ກ່ຽວຂ້ອງ):

_✓_ ການເຂົ້າເຖິງຫ້ອງປະຊຸມ

_✓_ ການເຂົ້າເຖິງຄອມພິວເຕີ ແລະ ເຄື່ອງພິມ

_✓_ ການເຂົ້າເຖິງອິນເຕີເນັດ

__ ການ​ເຂົ້າ​ເຖິງ​ໂທລະ​ສັບ​

_✓_ ການ​ເຂົ້າ​ເຖິງ​ຕູ້​ສໍາ​ລັບ​ການ​ເກັບ​ຮັກ​ສາ​ຂອງ​ແຟ້ມເອກະສານໂຄງຮ່າງການຄົ້ນຄວ້າວິໄຈ

3. REC ມີພະນັກງານບໍລິຫານທີ່ຖືກມອບໝາຍໃຫ້ REC ແມ່ນບໍ?

_✓_ ແມ່ນ __ ບໍ່

ຖ້າແມ່ນ: ເປັນພະນັກງານເຕັມເວລາບໍ? __ ແມ່ນ _✓_ ບໍ່

ເປັນພະນັກງານເຄິ່ງເວລາແມ່ນບໍ? _✓_ ແມ່ນ __ ບໍ່

ປະລິມານວຽກຂອງ REC

ຈໍາ​ນວນ​ສະ​ເລ່ຍ​ຂອງໂຄງຮ່າງການຄົ້ນຄວ້າວິໄຈທີ່ພິຈາລະນາໃນແຕ່ລະປີ​? __100_____

ຈໍານວນສະເລ່ຍຂອງການທົດລອງທາງຄລີນິກທີ່ພິຈາລະນາໃນແຕ່ລະປີ? __30_____

ຈໍານວນສະເລ່ຍຂອງການສຶກສາທາງລະບາດວິທະຍາ/ທາງການສັງເກດທີ່ພິຈາລະນາໃນແຕ່ລະປີ? __30_____

ຫຼັງຈາກການກວດເບິ່ງແບບສັ້ນໆຂອງບັນທຶກກອງປະຊຸມ REC ສາມຄັ້ງຜ່ານມາ, ໃຫ້ປະກອບຕາຕະລາງຕໍ່ໄປນີ້ດ້ວຍ

ຕົວເລກສະເພາະ ຫຼື N/A (ບໍ່ມີຂໍ້ມູນ).

| ຕາຕະລາງປະລິມານວຽກຂອງ REC | ກອງປະຊຸມ ຄັ້ງທີ 1 | ກອງປະຊຸມ ຄັ້ງທີ 2 | ກອງປະຊຸມ ຄັ້ງທີ 3 |
| --- | --- | --- | --- |
| ໄລຍະເວລາຂອງກອງປະຊຸມ | ກຸມພາ | ເມສາ | ມິຖຸນາ |
| ຈໍານວນໂຄງຮ່າງການຄົ້ນຄວ້າວິໄຈໃໝ່ທີ່ພິຈາລະນາໂດຍຄະນະກຳມະການເຕັມ | 6 | 12 | 6 |
| ຈຳນວນໂຄງຮ່າງການຄົ້ນຄວ້າວິໄຈທີ່ບໍ່ໄດ້ຮັບອະນຸມັດ | N/A | N/A | N/A |
| ຈຳນວນໂຄງຮ່າງການຄົ້ນຄວ້າວິໄຈໃນການພິຈາລະນາແບບຕໍ່ເນື່ອງ ທີ່ໄດ້ຮັບອະນຸມັດ ຈາກ ການພິຈາລະນາ ແບບເລັ່ງລັດທີ່ລາຍງານໃຫ້ REC | N/A | 1 | N/A |
| ຈໍາ​ນວນ​ໂຄງຮ່າງການຄົ້ນຄວ້າວິໄຈໃນການພິຈາລະນາແບບຕໍ່ເນື່ອງທີ່ໄດ້ຮັບການພິຈາລະນາໂດຍຄະນະກຳມະການເຕັມ | N/A | N/A | N/A |
| ຈຳນວນ ການປັບປຸງແກ້ໄຂທີ່ອະນຸມັດ ໂດຍການພິຈາລະນາ ແບບເລັ່ງລັດທີ່ ຖືກ ລາຍງານໃຫ້ REC | N/A | N/A | N/A |
| ຈໍາ​ນວນ​ການປັບປຸງແກ້ໄຂທີ່ພິຈາລະນາ​ໂດຍ​ຄະ​ນະ​ກໍາ​ມະການ​ເຕັມ​ | N/A | N/A | N/A |
| ຈໍານວນປະຕິກິລິຍາທາງລົບທີ່ພິຈາລະນາໂດຍຄະນະກໍາມະການເຕັມ | N/A | N/A | N/A |
