# Supplementary material for: Evaluating research ethics committees in Vietnam and Laos: Results of a validated self-assessment tool
Source: PLoS One. 2024 Aug 22;19(8):e0309084. doi: 10.1371/journal.pone.0309084 (PMC11340885; doi:10.1371/journal.pone.0309084)
Supplement: Attachment — Submitted filename: 1. Response to Reviewers.docx [file pone.0309084.s008.docx]

**Evaluating Research Ethics Committees in Vietnam and Laos: Results of a Validated Self-Assessment Tool**

**We are pleased that some of the reviewers recognize the value of thus work for publication. We thank the journal for the opportunity to make it stronger based on the comments below**

**PLOS ONE Reviewer Comments**

**Reviewer 1:**

1. Dear Authors,

Thank you for referring to my comments and making changes in the manuscript. One additional request: Please include a reference to the Supplementary Materials in the manuscript text. I recommend acceptance of the paper.

References to the Supplementary Materials have been added to lines 89, 121, and 189. Additionally, a “Supporting Information” section has been added to the end of the manuscript detailing the supporting information with captions.

**Reviewer 3:**

The background section lacks enough content on the status of RECs in the two countries, other the LMICs and various tools available for evaluation of RECs.

Peer-reviewed literature about research ethics in Vietnam and Laos are sparse. Some information regarding the oversight of clinical research in each country has been added to lines 72 - 77. As stated in line 79, this is the first study characterizing the RECs in Vietnam and Laos.

An extra source on RECs in other LMICs was added to lines 63 - 66 to highlight opportunities for improvements in training/education of research ethics.

A sentence explaining our choice of survey for this study was added to the Methods in lines 115 - 119.

**Reviewer 4:**

1. The inclusion and exclusion criteria in methodology needs further elaboration. Line 86: "active involvement" seems to be an inclusion criterion; however, it would be better if you could point out what that concretely means. Try to name your exclusion criteria, if any, to strengthen the section.

More precise inclusion and exclusion criteria have been added to lines 93 - 96.

2. Line 259, 260: Assessing the functionality of RECs based solely on the infrastructures or the standard operations is not unfortunately possible. Other factors such as REC members' experience or skills may play vital roles.

This sentence has been modified to take this comment into account in lines 275 - 276.

3. The comparisons with RECs in the Middle East, Africa, and South Asia at discussion are insightful, but they require more detailed data to be fully convincing. While relevant studies have been cited and provided survey data, adding specific performance metrics and more detailed statistical comparisons would strengthen these sections. (Statistically, if you want to justify that a country performed better, you cannot just compare the scores.)

While statistical analysis to compare the validated survey results was not performed in this study, the discussion of the literature on this topic has been modified based on these comments in lines 296 - 301.

4. Most of the references are older than 5 years. It would be great if you could check and add newer references.

5 additional references published within the last 5 years were added while addressing all reviewer comments.
