# Supplementary material for: Evaluating research ethics committees in Vietnam and Laos: Results of a validated self-assessment tool
Source: PLoS One. 2024 Aug 22;19(8):e0309084. doi: 10.1371/journal.pone.0309084 (PMC11340885; doi:10.1371/journal.pone.0309084)
Supplement: Attachment — Submitted filename: 1. Reviewer Comments WTL.docx [file pone.0309084.s007.docx]

**Evaluating Research Ethics Committees in Vietnam and Laos: Results of a Validated Self-Assessment Tool**

**PLOS ONE Reviewer Comments**

We thank the reviewers for recognizing our work to be an “important topic”
“compelling subject matter” and “scientific value”. Below is the point-by-point response to the valuable comments. Thank you for helping us improve our report.

**Reviewer 1:**

1. It is worth adding (e.g. as supplementary material) the form used to evaluate the performance of research ethics committees. The authors indicate that the base reference is an article by Sleem (2010) but adding a direct form that was used will increase the readability of the article.

A copy of the survey created by Sleem et al. and used in this study has been added to the supplementary materials.

2. I would like to ask the authors to add additional explanations on how the institutions and their representatives were selected and who was responsible for conducting the evaluation. What were the criteria for inclusion and exclusion of institutions? Please specify what is meant by the term "potential representatives".

We have clarified that this study is based upon a convenience sample of institutions and that the Vietnam National University – University of Medicine and Pharmacy and the Laos Ministry of Health aided us in the recruitment of the participating institutions (lines 82 - 87). This has also been added as a limitation to this study (line 297). Of note there are only 2 review boards in Laos and both did participate.

We have clarified the inclusion criteria (line 86).

We have clarified that specifically members of the REC at each institution agreed to participate in the study and filled out the survey (line 98, line 100, line 114)

3. Does the study have a research protocol, and if so, has it had prior registration?

This study was deemed exempt by our institutions institutional review board. Our outcome letter has been provided with our submission.

4. In the description of the methods, the authors mention that the maximum possible score is 200. However, it is not clear what this means. Since in results the number of points obtained is a key part, it is necessary to add an explanation of what this means. Please enrich the manuscript with more data on the tool used.

This study utilized a survey created by Sleem et al. (as referenced in the manuscript) which indicates that the survey design was based upon internal guidelines and standards for RECs. An explanation regarding the maximum score was added to line 124.

5. line 117-119 p. 4: The article should describe in detail how the statistical analysis was carried out - what statistical methods were used for comparison.

A more detailed explanation of the statistical methods, including statistical test and software employed, has been added to lines 156 – 162.

6. line 121-122 p.4: “The following general characteristics for analysis were used based on prior studies using this tool in other LMICs.” Citations needed.

A citation has been added to this sentence on line 165.

7. Data are presented at the research ethics committee level. However, the number of respondents within each committee is not reported. Was one representative selected within each institution?

It has been specified that one REC member from each institution completed the survey (line 114).

8. Data on what the responses to each question were within the domains of the survey should also be included in the manuscript (or in supplementary material). In addition, it would be useful to give a more detailed explanation of what each domain means.

Our raw, untranslated and scored survey responses have been provided as supplementary material.

Furthermore, a paragraph detailing the contents of each survey domain has been provided on lines 132 - 154.

9. It is not clear if the survey was anonymous. I can't find any information about the institutions that participated in the survey, does this mean that the data was not collected?

The survey was not anonymous, although the institutions were deidentified during analysis. This clarification has been added to line 124.

The names of the institutions that participated in this study has been added to lines 90 - 98.

10. The conclusions presented in lines 212-217 (page 8) seem too far-fetched. Does the existence of standard operating procedures and infrastructure for declaring conflicts of interest mean effective operation? In this situation, it is only a premise, as we do not know how it is used in practice. Correction of that sentences is needed. In excerpts on lines 212-233 on page no. 8, additional references should be inserted to support the authors' statements.

We have modified our statements in lines 258 - 260 and line 304 to more appropriately characterize our findings.

References have been added to lines 265, 271, 276 to bolster some of the suggestions made.

**Reviewer 2:**

The absence of documented ethical clearance or ethical review for the study.

As clarified in line 82 and as demonstrated by the outcome letter included in our submission, this study was deemed exempt by the institutional review board at our institution.

Moreover, the opacity surrounding the number of subjects interviewed, coupled with the absence of the questionnaire (at least in the English version) and an adequate description exposition of the tool employed, renders the methodology incompletely documented. Likewise, insufficient elucidation of the translation and adaptation process exacerbates the methodological ambiguity.

A copy of the survey created by Sleem et al. and used in this study has been added to the supplementary materials.

As stated in our methods section, an authorized translation service called Stepes was utilized to translate the survey in Vietnamese and Laotian. The certifications for this translation have been included in our supplemental materials. Furthermore, these methods were reviewed by the institutional review board at our institution.

Lastly, the conclusions drawn in the article appear unsubstantiated by the evidence presented. For instance, the extrapolation of 'effective functioning' solely from the mere presence of standard operating procedures and infrastructure for declaring conflicts of interest seems unwarranted.

We have modified our statements in lines 258 - 260 and 304 to more appropriately characterize our findings.
